# The role of salivary metabolomics in chronic periodontitis: bridging oral and systemic diseases

**DOI:** 10.1007/s11306-024-02220-0

**Published:** 2025-02-07

**Authors:** Jawaher Albahri, Heather Allison, Kathryn A. Whitehead, Howbeer Muhamadali

**Affiliations:** 1 Centre for Metabolomics Research, Department of Biochemistry, Cell and Systems Biology, Institute of Systems, Molecular and Integrative Biology, University of Liverpool, Liverpool, L69 7ZB UK; 2 Department of Pharmaceutical Chemistry, College of Pharmacy, King Khalid University, Abha, 62529 Saudi Arabia; 3 Microbiology at Interfaces, Department of Life Sciences, Manchester Metropolitan University, Chester St, Manchester, M1 5GD UK; 4 Institute of Infection, Veterinary and Ecological Sciences, University of Liverpool, Liverpool, UK

**Keywords:** Metabolites, Chronic periodontitis, Oral cavity, Bacteria, Biomarkers

## Abstract

**Background:**

Chronic periodontitis is a condition impacting approximately 50% of the world’s population. As chronic periodontitis progresses, the bacteria in the oral cavity change resulting in new microbial interactions which in turn influence metabolite production. Chronic periodontitis manifests with inflammation of the periodontal tissues, which is progressively developed due to bacterial infection and prolonged bacterial interaction with the host immune response. The bi-directional relationship between periodontitis and systemic diseases has been reported in many previous studies. Traditional diagnostic methods for chronic periodontitis and systemic diseases such as chronic kidney diseases (CKD) have limitations due to their invasiveness, requiring practised individuals for sample collection, frequent blood collection, and long waiting times for the results. More rapid methods are required to detect such systemic diseases, however, the metabolic profiles of the oral cavity first need to be determined.

**Aim of review:**

In this review, we explored metabolomics studies that have investigated salivary metabolic profiles associated with chronic periodontitis and systemic illnesses including CKD, oral cancer, Alzheimer’s disease, Parkinsons’s disease, and diabetes to highlight the most recent methodologies that have been applied in this field.

**Key scientific concepts of the review:**

Of the rapid, high throughput techniques for metabolite profiling, Nuclear magnetic resonance (NMR) spectroscopy was the most applied technique, followed by liquid chromatography-mass spectrometry (LC-MS) and gas chromatography-mass spectrometry (GC-MS). Furthermore, Raman spectroscopy was the most used vibrational spectroscopic technique for comparison of the saliva from periodontitis patients to healthy individuals, whilst Fourier Transform Infra-Red Spectroscopy (FT-IR) was not utilised as much in this field. A recommendation for cultivating periodontal bacteria in a synthetic medium designed to replicate the conditions and composition of saliva in the oral environment is suggested to facilitate the identification of their metabolites. This approach is instrumental in assessing the potential of these metabolites as biomarkers for systemic illnesses.

## Introduction

Chronic periodontitis is one of the most prevalent diseases in the world. According to a systemic analysis of the global burden of oral diseases that covers around three decades from 1990 to 2017, periodontitis in its severe form presented in about 10% of the worldwide population, with a high occurrence in people between 60 and 64 years of age (Bernabe et al., 2020). In contrast, approximately 50% of adults have shown signs of moderate periodontitis (Eke et al., 2018). Chronic periodontitis is an infectious disease resulting in the progressive inflammation of the periodontium, the tissues that surround and support the teeth, due to chronic responses between the host immune system and bacterial colonisation, which results in a biofilm that can extend to the root of the teeth (Wang et al., 2019). As a result, the range of bacteria that colonise the periodontium and the teeth roots can come in direct contact with the circulatory system.

Since the onset of chronic periodontitis results in an increase in the amount of bacterial burden within the oral cavity, it can initiate or exacerbate an inflammatory immune response leading to, or enhancing several systemic diseases (Genco & Sanz, 2020), e.g. oral cancer (Lim et al., 2018), Alzheimer’s disease (Gaur & Agnihotri, 2015), Parkinson’s disease (Olsen et al., 2020), diabetes (Chapple et al., 2013), and chronic kidney disease (Lapo et al., 2021).

This review aims to highlight the recent methodologies that have been used to investigate either the salivary or the gingival crevicular fluid (GCF) metabolome and its association with chronic periodontitis and systemic diseases. It is worth noting that metabolomics serves as the keystone for designing biomolecule-specific biosensors, which can be used for the detection and identification of bacterial-specific metabolites (biomarker) in saliva. The discovery of such biomarkers would be a step forward to use saliva and/or GCF as targeted biofluids for the early diagnosis of systemic disease in patients who have the potential to develop such illnesses.

## Bacteria found in the oral cavity

### Bacteria in the healthy mouth

The oral cavity hosts a diverse microbial community including around 700 species that generally exist in a balanced, symbiotic relationship with the host and contributes to essential functions such as maintaining oral health (Paster et al., 2006). Such microbiota include Gram-positive cocci (Streptococcus mutans, Streptococcus mitis, Streptococcus sanguinis, Streptococcus oralis, Rothiadentocariosa, Staphylococcus epidermidis), Gram-positive bacilli (Actinomyces viscosus, Actinomyces israelis, Actinomyces gerencseriae, Clostridium spp.), and Gram-negative cocci (Veillonella parvula, Neisseria spp.) (Listgarten, 1976). However, this relationship is not exclusively symbiotic. In healthy individuals, such bacterial species exist in a state of commensalism, where they benefit from the environments of the oral cavity without significantly impacting the host health (Lof et al., 2017). Therefore, such bacteria could be considered the first line of protection against pathogenic microbes such as bacteria, viruses, and fungi (Shakya et al., 2018). This commensal state is dynamic, with the microbiota continuously adapting to environmental shifts in the mouth that are attributed to factors such as age, pregnancy, alcohol, smoking, medications, genetic predisposition, changes in diet, hygiene practices, and saliva composition (Marsh, 2018). The overall microbial community remains balanced as long as these factors promote stability, allowing the oral microbiota to support health by contributing to immune modulation and preventing pathogenic overgrowth. The normal oral microbiota, when in balance, effectively inhibits pathogen growth. In addition, they can compete for the attachment sites inside the mouth, such as teeth and gingiva, further preventing their colonisation by foreign microbes (Jenkinson & Lamont, 2005). However, this equilibrium can be disrupted under certain conditions, leading to opportunistic interactions that can affect oral and systemic health (Brennan & Garrett, 2019). Factors such as weakened host defences, poor oral hygiene, or changes in pH can prompt some commensal bacteria to behave opportunistically, contributing to disease states (Kilian et al., 2016; Marsh et al., 2014; Wu et al., 2016). In a compromised environment, certain bacteria may proliferate or express virulence factors that drive the development of conditions like periodontitis (Santacroce et al., 2023). Thus, the relationship between the host and oral microbiota encompasses a range of interactions, such as symbiotic, commensal, and opportunistic, shaped by the dynamic and complex ecosystem of the oral cavity (Samaranayake & Matsubara, 2017). Recognising this complexity is crucial for understanding how microbial communities contribute to health and disease in the oral environment. Streptococcus spp. are considered the main genus of bacteria that constitute the oral microbiota, and these can be detected in the neonatal mouth within the first few hours of birth for example, Streptococcus salivarius, has been identified within 8 h after an infant’s birth (Rotimi & Duerden, 1981). Multispecies synergy develops through diverse interspecies interactions, manifesting in providing substratum for attachment and colonisation, nutritional cross-feeding between species, and collaboratively metabolising complex substrates (Hajishengallis & Lamont, 2016; Lamont & Hajishengallis, 2015; Lamont et al., 2018; Short et al., 2014). However, the interrelationship between the different species is complex and could be multifaceted. Streptococcus gordonii, Streptococcus parasanguinis, S. sanguinis, S. oralis, and S. mitis are abundant colonisers that express various adhesins for the salivary pellicle receptors that coat tooth surfaces (Nobbs et al., 2009). Such microorganisms are not considered pathogenic on their own, and they can reduce the pathogenicity of other organisms (Lamont et al., 2018; Whitmore & Lamont, 2011). S. gordonii, S. sanguinis, and S. oralis have the potential to protect against dental caries as they are antagonistic toward S. mutans (Cheng et al., 2018; Thurnheer & Belibasakis, 2018). Furthermore, Streptococcus cristatus can reduce the pathogenicity of Porphyromnas gingivalis by suppressing virulence gene expression (Ho et al., 2017). In contrast, the coexistence of P. gingivalis wih *S. gordonii* exacerbates the alveolar bone loss (Daep et al., 2011). Furthermore, the streptococcal metabolite 4-aminobenzoate/para-aminobenzoic acid (PABA) was found to enhance the attachment of *P. gingivalis*, and reduces its virulence activity by suppressing the production of extracellular polysaccharides (Kuboniwa et al., 2017). *S. gordonii and S. parasanguinis* were found to enhance the pathogenicity of *Aggregatibacter actinomycetemcomitans* (Duan et al., 2016). Moreover, *S. gordonii*,* S. oralis*,* and S. sanguinis* were found to promote the virulence activity of *Candida albican* (Bertolini et al., 2015). Hence, when their synergistic effect manifested in enhancing the virulence activity of the keystone pathogens, the normal oral microbiota are sometimes referred to as accessory pathogens (Shaikh et al., 2018; Whitmore & Lamont, 2011).

### The complex theory

**Fig. 1:**
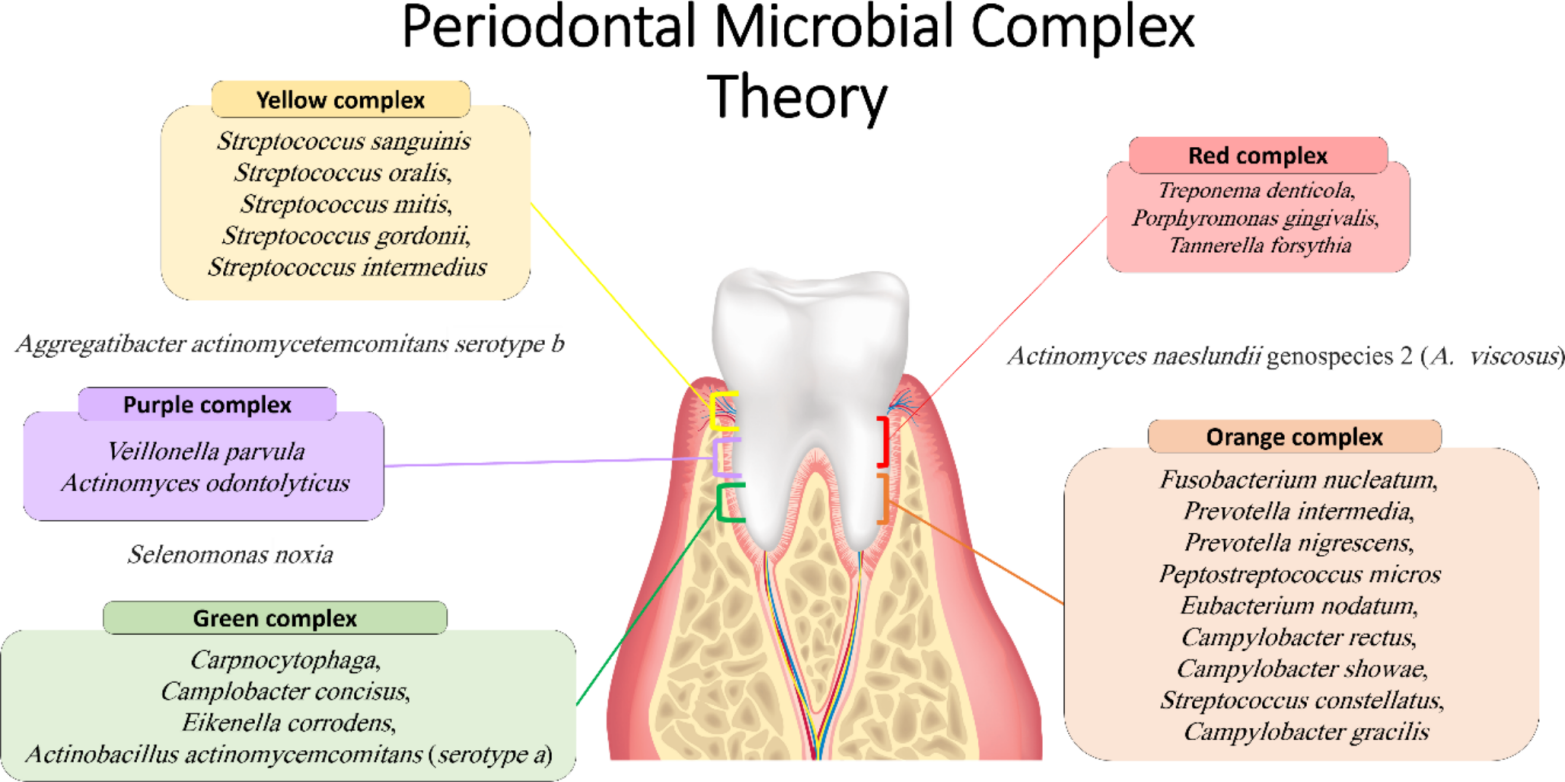
Subgingival plaque complex theory. Different coloured backgrounds represent each of the 5 different complexes. Adapted from Socransky et al., 1998.

In order to study and classify bacteria involved in the development of periodontal plaque (biofilm) and associated with periodontal diseases, Sigmund Socransky and his colleagues developed the complex theory (Socransky et al., 1998) (Fig. 1).

This theory classifies bacteria involved in periodontitis according to their pathogenicity and role in biofilm development into five colour-labelled groups: red, orange, yellow, green, and purple which became known as complexes. In addition to the coloured complexes, *Aggregatibacter actinomycetemcomitans* serotype b, *Selenomonas noxia* and *Actinomyces naeslundii* genospecies 2 (*A. viscosus*) were stated as outliers due to their limited relation to each other and the five significant complexes (Socransky et al., 1998).

The commensal bacteria, typically found in healthy oral cavities, help the immune system by preventing pathogenic bacterial colonisation and invasion of human body systems (Gao et al., 2018). Conversely, it is assumed that under certain circumstances, they could synergistically assist the pathogenesis of chronic periodontitis by enhancing the virulence of pathogens through a network of interactions (El-Awady et al., 2019). However, many of these interactions are complex and not yet very well understood. It has been suggested that such commensal bacteria could assist the pathogenesis of chronic periodontitis through several roles, such as enhancing the pathogens’ adherence by providing an attachment substrate (Whitmore & Lamont, 2011), destabilising the innate immune system to maintain the periodontal pockets colonisation (Guan et al., 2008), releasing pro-inflammatory cytokines to initiate periodontium devastation (Ramadan et al., 2020), producing a leukotoxin for gingival epithelial cells apoptosis (Henderson et al., 2010), or facilitating the coaggregation of the colonising pathogens (Settem et al., 2012). Besides their roles in the pathogenesis of chronic periodontitis, many of such pathogens have been reported to be associated with other systemic diseases. Bastos et al. compared chronic periodontitis in 47 patients with CKD and 19 participants without systemic illnesses, to identify the periodontic pathogens within the subgingival plaque (Bastos et al., 2011). The results showed that *P. gingivalis*,* Tannerella forsythia*, and *Treponema denticola* were the main periodontal pathogens identified in the CKD patients. In terms of Alzheimer’s disease, Leblhuber et al. examined the alveolar fluid of 20 individuals with primary degenerative dementia to also identify periodontal pathogens. The study identified *T. forsythia* and *T. denticola* as the predominant pathogenic strains (Leblhuber et al., 2020). Furthermore, *P. gingivalis* was found the most prevalent pathogen in the oral cancer (Lim et al., 2018), Parkinson’s diseases (Olsen et al., 2020), and diabetes (Aemaimanan et al., 2013) (Table 1).

**Table 1:** Chronic periodontitis associated pathogens that have been detected with certain systemic diseases

Systemic disease		Oral microbial biomarker candidate	Sample origin	Reference
	**Oncological disorders**			
Oral cancer		*Porphyromonas gingivalis*	Oral rinse	(Lim et al., 2018)
	**Neurological and cognitive diseases**			
Alzheimer’s disease		*Porphyromonas gingivalis*, *Aggregatibacter actinomycetemcomitans*, *Fusobacterium nucleatum*	Subgingival plaque	(Maurer et al., 2018)
		*Tannerella forsythia*, *Treponema denticola*	GCF	(Leblhuber et al., 2020)
Parkinson’s disease		*Porphyromonas gingivalis*	Blood	(Adams et al., 2019)
	**Chronic disease**			
Chronic kidney disease		*Porphyromonas gingivalis*, *Tannerella forsythia*, *Treponema denticola* *Candida albicans*	Subgingival plaque	(Bastos et al., 2011)
Diabetes		*Porphyromonas gingivalis*, *Tannerella forsythia*, *Treponema denticola*	Subgingival plaque	(Aemaimanan et al., 2013)

### Changes in bacterial species with the onset of chronic periodontitis

Chronic periodontitis is progressively caused by the imbalance of the oral microbial community, which is developed over an expanded period, resulting in a microbial-complex shift that is mainly manifested with bacteria associated with the orange complex (*Prevotella intermedia*,* Prevotella nigrescens*,* Peptostreptococcus micros*,* Eubacterium nodatum*,* Campylobacter rectus*,* Campylobacter showae*,* Streptococcusc constellatus*,* Campylobacter gracilis*, and *Fusobacterium nucleatum*), to the red complex (*T. forsythia*,* T. denticola*, and *P. gingivalis*) (Shaikh et al., 2018). Subgingival bacteria are considered the main bacterial agents of chronic periodontitis since they affect the metabolic process of the normal microbiota, consequently exacerbating the flow of GCF (Subbarao et al., 2019). The development of the red complex is mainly influenced by the presence of the orange-complex and thus the higher orange complex colonisers lead to red complex colonisation (Mohanty et al., 2019).

Although GCF and saliva play a protective role against oral pathogens due to their antimicrobial components, such as hydrogen peroxide, lactoferrin, and lysozymes (Vila et al., 2019), they are rich in nutrients that can promote the growth of anaerobic bacteria (Kilian et al., 2016). *P. gingivalis* has shown nutritional interdependency with *T. denticola* and they have been reported to have a crucial role in the destruction of periodontal tissue due to their ability to release several outer-membrane related enzymes called proteinases such as arginine- and lysine-specific cysteine proteases, and a chymotrypsin-like serine protease, which are also found in the outer-membrane-derived blebs that are released (Holt & Ebersole, 2005). *T. forsythia* is essential for the presence of *P. gingivalis*, and the detection of *P. gingivalis* in the absence of *T. forsythia* seems unlikely (Yang et al., 2004). *T. forsythia* has a role in providing a favourable microbial environment within the biofilm via releasing proteinases that degrade the extracellular biomolecules, which impair host response factors (Tanner et al., 1986).

A number of other members of the microbiota have also been reported to have a cooperative role in chronic periodontitis pathogenesis, such as those of the *Streptococcus anginosus* group (*S. anginosus*,* S. constellatus*,* S. intermedia*), *Streptococcus mitis* group (*S. gordonii*,* S. sanguinis*,* S. oralis*), *A. israelii*,* A. actinomycetemcomitans*,* F. nucleatum*, and *P. intermedia* (Hickey et al., 2020). Therefore, chronic periodontitis occurrence and progression is believed to be the result of a multifaceted synergy among various microorganisms and a state of dysbiosis (Hickey et al., 2019).

Recent advancements in next-generation sequencing (NGS) have transformed our understanding of the bacterial diversity associated with chronic periodontitis. Unlike the traditional Sanger sequencing method, NGS performs high-throughput sequencing of hundreds of fragments across large genomic regions enabling comprehensive profiling of the oral microbiome, revealing complex microbial shifts with the onset of the disease (Kilian et al., 2016; Koboldt et al., 2013). NGS-based studies have confirmed a significant increase in the prevalence of red-complex species with disease onset and have revealed roles of additional taxa, such as *Filifactor alocis*, which appear to contribute to disease progression (Griffen et al., 2012). Using 454-pyrosequencing of 16 S rRNA gene libraries and quantitative PCR, *Spirochetes*, *Synergistetes*, *Firmicutes*, and *Chloroflexi* were found as dominant genera in the subgingival plaque in chronic periodontitis, while *Actinomyces* were found at higher proportions in healthy subjects (Abusleme et al., 2013). In patients with chronic periodontitis, NGS studies identified *Porphyromonas*, *Treponema*, *Fusobacterium*, *Tannerella*, *Prevotella*, and *Filifactor* as the most prevalent genera in subgingival plaque (Han et al., 2017; Liu et al., 2018; Shi et al., 2015), as well as in both subgingival plaque and saliva (Chen et al., 2018) before undergoing scaling and root planning (SRP) (Pihlstrom et al., 2005). In contrast, following SRP treatment, an increase was observed in the relative abundance of four genera: *Streptococcus*, *Actinomyces*, *Rothia*, and *Veillonella*. Furthermore, there was a distinct separation of such microbiota between saliva and plaque samples as they were found at lower alpha diversity i.e. less richness and evenness in saliva than in plaque samples. However, findings on alpha diversity shifts post-treatment are conflicting; while one study reported a decrease in diversity in both subgingival plaque and saliva samples (Belstrøm et al., 2018), another indicated that alpha diversity in saliva remains stable following treatment (Yamanaka et al., 2012). Interestingly, a significant overall correlation was reported between periodontitis-associated species in subgingival plaque and saliva, suggesting potential reflection of the subgingival colonisation (Belstrøm et al., 2018). Using Miseq Sequencing of 16S rRNA genes, Wei et al. reported change in diversity of both buccal and subgingival plaque microbiota where Veillonella, Treponema, Filifactor, Fretibacterium, Peptostreptococcaceae_[XI][G-6], Peptostreptoc occaceae_[XI][G-5], Bacteroidetes_[G-5], Bacteroidetes_[G-3], Peptostreptococcaceae_[XI][G-4], and Peptostreptococcaceae_[XI][G-2] exhibited significant increase both in buccal and subgingival plaque samples in periodontitis compared with healthy subjects (Wei et al., 2019). In a more recent study, using 16 S rRNA gene metagenomic sequencing, the salivary microbiome showed differences between periodontitis and healthy subjects. *P. gingivalis* exhibited the highest relative abundance in periodontitis patients among the red complex pathogens in addition to *Synergistetes*, *Synergistia*, *Synergistales*, *Synergistaceae*, *Fretibacterium*, *Sinanaerobacter*, and *Filifactor* (Lee et al., 2023). Current literature indicates that no single microbial species can serve as a definitive biomarker for chronic periodontitis. Instead, this condition is associated with shifts in multiple species and complex microbial interactions influenced by factors like host defences and the oral health environment. Notably, these microbial changes are distinct from those seen in other polymicrobial infections, such as dental caries (Xiao et al., 2016), underscoring the unique progression of periodontitis. These specific characteristics should be carefully considered when developing therapeutic strategies for chronic periodontitis.

## Composition of fluids found in the oral cavity

In the oral cavity, there are two primary biofluids: saliva and GCF.

### Composition of saliva

Saliva is the prominent fluid in the mouth, which is a mixture of fluids secreted by salivary glands i.e. parotid glands, submandibular glands, and sublingual glands. In addition, it includes excretions and particles from tears, nasal and bronchial secretions, bacteria, residual food particles, and GCF (Navazesh, 1993). It is mainly composed of water, minerals, antimicrobial components, and enzymes such as alpha-amylase and lipase (Nassar et al., 2014). However, salivary production and the concentration of its components vary according to factors such as age (Cabras et al., 2009; Denny et al., 1991; Nassar et al., 2014), gender (Fenoll-Palomares et al., 2004), resting/stimulated state (Bhattarai et al., 2018), alcohol consumption or consuming food/drinks that reduce salivary pH (Klein et al., 2010). Therefore, it is crucial to take such factors into consideration when selecting the appropriate sample collection technique to achieve both high-quality data and to minimise unwanted variation.

### Gingival crevicular fluid composition

GCF is the biofluid that fills the periodontal pocket, and in healthy individuals, it is present in small amounts, but significantly rises during pathological conditions, e.g. inflammation (Taylor & Preshaw, 2016). The intricate composition of this substance primarily consists of serum, alongside locally produced elements including cellular elements (epithelial cells, leukocytes, and bacteria), electrolytes (sodium, potassium, phosphorus, fluoride, calcium, iodine and minerals) and organic compounds (carbohydrates, proteins, immunoglobulins, complement, cytokines, metabolic and bacterial products) (Lamster, 1997). It also includes enzymes such as proteolytic and hydrolytic enzymes of inflammatory cell origin, collagenases and related metalloproteinases (cysteine proteinases, aspartate proteinases, and serine proteinases) dipeptidyl peptidase (myeloperoxidase), aspartate aminotransferase and lactate dehydrogenase (Eley & Cox, 2003). The GCF plays a crucial role in preserving the integrity of the junctional epithelium and contributing to the antimicrobial defence mechanisms within the periodontium (Subbarao et al., 2019).

Although GCF and saliva have a defending role against oral pathogens (Vila et al., 2019), they are rich in nutrients that enhance bacterial growth, in particular cystine which creates an environment that supports the anaerobic growth of bacteria (Kilian et al., 2016). Given the specific growth requirements of subgingival bacteria, previous studies have utilised artificial saliva or GCF to emulate the native habitat required to facilitate the growth of these microorganisms (Hickey et al., 2019).

### Saliva for diagnostic purposes

In periodontitis, the structural integrity of the periodontal tissues is compromised because of chronic inflammation (Nanci & Bosshardt, 2006), leading to the formation of periodontal pockets extending apically from the cementoenamel junction (CEJ). This is the anatomical interface where the enamel of the tooth crown merges cementum, a calcified connective tissue covering the tooth root, making the pocket a reservoir for the GCF accumulation (Vandana & Haneet, 2014). Furthermore, the junctional epithelium becomes more permeable, and the GCF flow rate increases significantly during periodontitis (Barros et al., 2016). Such factors exacerbate the leakage of the GCF into saliva, making the saliva a target for detecting the subgingival pathogens and their toxins. Saliva has several advantages for diagnostic purposes over other human biofluids as it can be easily and rapidly collected (Prasad et al., 2016), using non-invasive techniques and it does not require special skills for its collection, making it convenient for both patients and staff (Celec et al., 2016). Moreover, the ease of storage for saliva in comparison to blood, is notable. Unlike whole blood, which necessitates initial processing before storage at -80 °C, saliva requires less intricate preparation. Therefore, designing a biosensor to detect the chronic periodontitis pathogen’s secondary metabolites (biomolecules) within saliva could provide a quick, easy, and accurate diagnostic tool for detecting a range of systemic diseases such as chronic kidney disease.

## An introduction to metabolomics

Metabolomics involves the comprehensive analysis of metabolites, which are the low molecular weight molecules found in a biological system, using a variety of analytical techniques (Klassen et al., 2017). Metabolomics analysis offers valuable biochemical insights that can aid in classifying a patient’s physiological or pathological condition. This information is instrumental for disease diagnosis and patient monitoring, enabling the identification of disease risk factors at a broader population level (Nicholson et al., 2012; Rathnayake et al., 2013). Due to their versatility, accessibility, and robustness, Nuclear magnetic resonance (NMR) spectroscopy, and Mass spectrometry (MS) coupled with chromatographic separation techniques, such as liquid chromatography (LC) and gas chromatography (GC), are known as the most popular analytical platforms used in the field of metabolomics (Marshall & Powers, 2017).

Metabolomics approaches employ a combination of analytical and computational techniques to detect, identify, and examine the significance of the metabolites present in complex mixtures (Weber et al., 2017). Given the broad range of metabolites within the metabolome, characterised by a range of chemical and physical characteristics, it is impractical to rely solely on a single analytical method for their detection. Thus, employing a strategy that combines multiple analytical platforms is paramount to achieve comprehensive coverage of the metabolites. In the field of salivary metabolomics, NMR and MS-based techniques are the most commonly used analytical platforms. However, the choice of metabolomics approach may vary based on the type and concentration of the metabolites of interest. Hence, a brief overview of these approaches is provided with definitions of the relevant terminologies used in the field of metabolomics (Table 2).

**Table 2:** Description of related metabolomics terminologies

Term	Definition	Reference
**Metabolite**	Small endogenous molecules which are formed during anabolism/catabolism processes such as amino acids, lipids, nucleotides, sugars, etc.	(Baghel et al., 2021)
**Metabolome**	Full set of metabolites that exist in a biological fluid/tissue sample at a particular physiological or developmental state.	(Goodacre, 2004; Viant et al., 2017)
**Metabolite profiling**	This is used interchangeably with metabolic profiling. It involves quantitative and qualitative detection of a group of compounds that are related to a particular pathway or a class of metabolites and their intermediates which have similar chemical properties within a biological system. Examples of such compounds/metabolites are carbohydrates, amino acids, etc.	(Fiehn, 2001; Goodacre et al., 2004)
**Targeted metabolomics/analysis**	Restricted quantitative and qualitative analysis of a specific set of metabolites with a previously known identity, or a particular metabolic pathway.	(Broadhurst et al., 2018; Fiehn, 2001; Goodacre et al., 2004)
**Untargeted metabolomics/analysis**	A semi-quantitative and global analysis approach that aims to measure the maximum number of metabolites. The chemical identity of the measured metabolites is not required to be known before data acquisition. Such results can be utilised for hypothesis generation and follow-up targeted studies.	(Broadhurst et al., 2018; Schrimpe-Rutledge et al., 2016)
**Metabolic fingerprinting**	This approach is a subdivision of the untargeted analysis. It aims to classify the samples according to their biological relevance/origin. Such techniques can be used for semi-quantitative analysis of the intracellular metabolites of an organism of interest.	(Ellis et al., 2007; Fiehn, 2001; Goodacre et al., 2004)
**Metabolic footprinting**	This is considered an untargeted approach. It focuses on analysing the exometabolome, i.e., extracellular metabolites that are excreted/secreted by a cell/system within the biological sample. This term also includes all the extracellular compounds, such as culturing media elements that had not been consumed by the microorganism.	(Ellis et al., 2007; Klassen et al., 2017)

### MS-based metabolomics to investigate chronic periodontitis

Mass Spectrometry (MS) technologies are robust analytical tools with multifaceted advantages in salivary metabolomics. They enable accurate identification and quantification based on mass-to-charge ratio (*m/z*), providing increased sensitivity and specificity (Zhu & Fang, 2013). MS techniques, especially tandem mass spectrometry (MS/MS), play a crucial role in unravelling the structural intricacies of salivary metabolites, and contribute significantly to understanding the complex biological composition of saliva (Grocholska et al., 2023). Various studies have used these powerful tools to explore the intricate metabolic signatures within saliva, particularly in the context of chronic periodontitis patients. Barnes et al. conducted an extensive untargeted metabolite profiling study on saliva, employing a comprehensive analytical approach (Barnes et al., 2011). The methodology involved two distinct ultra-high performance liquid chromatography-tandem mass spectrometry (UHPLC-MS/MS) injections, with the first specifically optimised for basic species and the second for acidic species identification; this was carried out in addition to GC-MS analysis. The findings of the study revealed that individuals with chronic periodontitis exhibited higher concentrations of 72 metabolites derived from the degradation of glycerophospholipids, triacylglycerols, polysaccharides and proteins compared to their healthy counterparts. The findings were suggested to be due to the increased activity in lingual lipase, α-amylase, elastases, amino peptidases, and metalloproteases. The elevated metabolites included lysolipids, monoacylglycerols, fatty acids (mainly dihomo-linolenate, arachidonate, docosapentaenoate, and docosahexaenoate), monosaccharides, oligosaccharides, dipeptides (mainly leucylisoleucine, threonylphenylalanine and serylisoleucine), cysteine, p-cresol sulfate, phenol sulfate, carnitine and 3-dehydrocarnitine.

In another salivary metabolomics profiling study, Kuboniwa et al. analysed whole saliva using GC coupled with time-of-flight mass spectrometry (GC-TOF/MS) (Kuboniwa et al., 2016). Multivariate regression analysis incorporating orthogonal projections to latent structures (OPLS) was undertaken to explore the correlation between the extent of periodontal inflammation, as indicated by the periodontal inflamed surface area (PISA), and the metabolic profiles observed in the saliva. Cadaverine, 5-oxoproline and histidine, were reported as the potential biomarkers for periodontitis. In a more targeted investigation, Balci et al. examined the levels of free amino acids in the saliva of periodontitis and healthy individuals using LC-MS-MS (Balci et al., 2021). The analysis revealed that citrulline, carnosine, methionine, glutamic acid, arginine, proline and tryptophan exhibited notably elevated levels in periodontitis patients compared to the control group.

Using MS techniques to scrutinise salivary metabolomics, an investigation conducted by Rodrigues et al. focused on the analysis of GCF in periodontitis patients (Rodrigues et al., 2021). GC-MS was employed to assess potential biomarkers associated with chronic periodontitis in participants aged ≥ 65 years. Subsequently, Partial Least Squares Discriminant Analysis (PLS-DA) was employed to discriminate between healthy and chronic periodontitis groups. This study identified several potential biomarkers indicative of chronic periodontitis, however, 5-aminovaleric acid and serine emerged as particularly noteworthy candidates, which was attributed to their significantly elevated concentrations within the GCF of chronic periodontitis patients. These findings were in agreement with prior research of Ozeki et al., who also identified 5-aminovaleric acid as a biomarker indicative of chronic periodontitis within the GCF (Ozeki et al., 2016).

While these studies provide valuable insights into salivary metabolite profiles, several limitations should be considered. The cross-sectional design of some studies restricts the ability to draw definitive conclusions about causal relationships between observed metabolic changes and disease progression. Additionally, the focus on older adults in certain studies may limit the generalisability of findings to younger populations, emphasizing the need for further research to validate these biomarkers across different age groups and demographics. Collectively, these limitations highlight the importance of standardised methodologies, larger and more diverse cohorts, and longitudinal designs to enhance the reliability and applicability of metabolomic biomarkers in periodontal disease research.

### Spectroscopy-based metabolomics to investigate chronic periodontitis

Spectroscopy techniques play a pivotal role in advancing our understanding of chronic periodontitis through salivary metabolomics, providing valuable insights into the molecular signatures and metabolic changes associated with this condition. Studies on saliva from chronic periodontitis patients using NMR spectroscopy have emerged with such techniques becoming predominant analytical platforms, signifying their widespread utilisation for exploring metabolic profiles associated with periodontitis. NMR spectroscopy is a robust and highly reproducible technique based on the principles of nuclear spin and magnetic resonance as it involves the interaction of atomic nuclei with an external magnetic field and radiofrequency radiation (Wishart, 2008). It can provide detailed information about the molecular structure of the metabolites with minimal sample preparation, and unlike GC-MS, it does not require time-consuming chemical derivatisation steps. Nevertheless, NMR has constraints when compared to MS, mainly due to its lower sensitivity, specifically, the detection limit for NMR resides in the low micromolar range, whereas for MS, it is within the picomolar range (Emwas et al., 2013; Wolfender et al., 2015). Moreover, it has low selectivity, which could be noted from the overlapping peaks of the various metabolites. Although it can detect a wide range of novel molecules, it is not suitable for detecting inorganic ions (Guennec et al., 2014).

### ^1^H-NMR

Proton nuclear magnetic resonance (^1^H-NMR) spectroscopy operates by exposing the sample to a strong magnetic field and applying radiofrequency radiation, which induces resonance in protons at frequencies determined by their chemical environment, resulting in an NMR spectrum. This provides detailed information about the number, chemical environment, and interactions of hydrogen atoms within the molecule (Sahoo et al., 2020). In research carried out by Singh et al., ^1^H-NMR spectroscopy was used to detect and identify potential biomarkers associated with chronic periodontitis in whole saliva collected from 114 participants (Singh et al., 2017). Following this, PLS-DA was implemented to distinguish individuals affected by chronic periodontitis from healthy individuals. The findings indicated that glutamate, valine, succinate, alanine, lactate, leucine, and pyruvate were elevated, demonstrating significant associations with chronic periodontitis within the examined participant group. Similarly, García-Villaescusa et al. investigated whole saliva samples obtained from 130 participants, employing ^1^H-NMR spectroscopy, followed by PLS-DA (García-Villaescusa et al., 2018). The primary objective was to distinguish between individuals with chronic periodontitis and those in a healthy state. The analysis revealed a significant upregulation of nine metabolites, namely 4-aminobutyrate, butyrate, caproate, choline, isocaproate, isoleucine, isopropanol, isovalerate and methanol, in chronic periodontitis patients. Concurrently, five metabolites including lactate, proline, sucrose and sucrose-glucose-lysine were found to be downregulated in individuals affected by chronic periodontitis.

In a different study, Romano et al. used ^1^H-NMR spectroscopy for the characterisation of potential biomarkers associated with chronic periodontitis within whole saliva collected from a cohort of 22 participants (Romano et al., 2019). After the spectral acquisition, Principal Component Analysis (PCA) followed by canonical correlation analysis (CCA) was implemented to discriminate individuals suffering from chronic periodontitis from their healthy counterparts. The outcomes of the investigation revealed isoleucine, phenylalanine, proline, tyrosine and valine as salivary biomolecules exhibiting upregulated levels which significantly correlated with chronic periodontitis patients, in addition to th downregulation of lactate and pyruvate. Notably, three of these identified metabolites, isoleucine, lactate (García-Villaescusa et al., 2018) and valine (Singh et al., 2017), were consistent with findings from previous studies utilising the same ^1^H-NMR technique for chronic periodontitis diagnosis. The high reproducibility of NMR makes it the most applied analytical platform in salivary metabolomics studies. However, the limitations across the above studies reveal common methodological challenges that limit their broader applicability. The studies utilised relatively small sample sizes, which compromises the statistical power and generalisability of the findings. Additionally, each study excluded individuals with systemic conditions or other potential confounders, while controlling smoking was inconsistent, limiting the applicability of the results. Another limitation lies in the exclusive use of ¹H NMR spectroscopy, which, while effective, lacks the sensitivity of mass spectrometry to detect low-abundance metabolites, thus reducing the overall depth of metabolic profiling. Furthermore, the salivary sampling protocols, although well-standardised, in some studies, did not sufficiently account for individual variability in salivary flow rates and diurnal fluctuations, potentially introducing inconsistencies in themetabolite levels recorded.

### Vibrational spectroscopy

#### Raman spectroscopy

Vibrational spectroscopy involves studying the atomic oscillations within molecules upon interaction with electromagnetic radiation, using distinctive methodologies such as infrared (IR) and Raman spectroscopies (Sathyanarayana, 2015). These techniques are instrumental in providing crucial insights into the molecular structure and composition of substances through the analysis of their vibrational modes such as stretching, bending and rotating, allowing for simultaneous capturing of a wide range of biomolecules such as proteins, lipids, and polysaccharides (Siebert & Hildebrandt, 2008). Given their high throughput, robustness and reproducibility, coupled with minimal sample preparation requirements and the added benefit of in situ analysis with Raman spectroscopy, these vibrational techniques prove to be promising fingerprinting tools within the context of metabolomics studies (Pirutin et al., 2023).

In the exploration of saliva as a diagnostic biofluid for periodontitis, the study by Gonchukov et al. used Raman spectroscopy to analyse saliva samples collected from a cohort of 20 participants (Gonchukov et al., 2011). The study observed spectral shifts in four peaks within the spectral data of chronic periodontitis patients, specifically at 1155 cm^− 1^ and 1525 cm^− 1^, attributed to C-C and C = C vibrations, respectively. The presence of these peaks was correlated with the elevation in the overall salivary total antioxidants level as the periodontitis progressed. Notably, additional signal shifts at 1033 cm^− 1^ and 1611 cm^− 1^ were identified in the spectra of individuals with severe periodontitis, however, these shifts were not conclusively assigned to any specific functional group in the context of this study.

Utilising Surface-enhanced Raman spectroscopy (SERS), another study conducted a comparative analysis of sialic acid levels in saliva among 93 participants categorised into groups representing patients with periodontitis, gingivitis or individuals with healthy periodontal conditions (Hernández-Cedillo et al., 2019). Sialic acid, which has been reported previously as an inflammatory biomarker and is frequently elevated in plasma and serum in systemic conditions such as cardiovascular diseases, tumours, and diabetes (Cheeseman et al., 2021), was also found to be substantially elevated in this study, specifically in individuals diagnosed with periodontitis.

In a subsequent investigation conducted by Kriem et al., the differentiation of periodontal bacteria, namely *F. nucleatum*,* S. mutans*,* Actinomyces naeslundii*,* P. nigrescens*, and *Veillonella dispar* was undertaken using Confocal Raman Spectroscopy (CRM) (Kriem et al., 2020). This was followed by the implementation of a 2-way orthogonal Partial Least Square with Discriminant Analysis (O2PLS-DA). Distinct variations in band patterns among the five specified species were discerned, particularly within the spectral region spanning 700 cm⁻¹ to 900 cm⁻¹, corresponding to the ring breathing vibrations of nucleotides (DNA and RNA). Additionally, distinctions were observed in the spectral range of 1500 cm⁻¹ to 1625 cm⁻¹, which were attributable to variations in amino acid composition among the bacterial species. Interestingly, the five species exhibited a clustering pattern consistent with the microbial complexes theory that was elucidated by Socransky et al. (1998). Overall, the previous studies underscored the strengths of Raman techniques in offering high-resolution, label-free analysis with potential clinical applications. However, the limitations include the reliance of some studies on a single biomarker, which poses challenges in diagnosing polymicrobial infection-caused diseases such as periodontitis. Additionally, addressing the complexity of biofilms, managing confounding factors, and ensuring the generalisability of findings across diverse conditions remain significant hurdles. These limitations underscore the need for further methodological refinement and the integration of complementary techniques to enhance diagnostic accuracy and applicability.

#### Fourier transform infrared (FT-IR) spectroscopy

While Fourier transform infrared (FT-IR) spectroscopy is extensively utilised in metabolomics studies, its application in investigating salivary metabolic signatures, especially in the context of periodontitis, is limited. Notably, FT-IR has found broader application in studies related to other oral and systemic diseases. For instance, in a recent study by Gieroba et al., FT-IR coupled with Raman spectroscopy, was employed to characterize biofilms produced by cariogenic streptococci, including *S. mutans* (CAPM6067), *S. sobrinus* (CAPM 6070), *S. sobrinus / downei* (CCUG 21020), *S. sanguis* (ATCC 10556), and *S. sobrinus* (DSMZ 20381) (Gieroba et al., 2020). The results revealed that biofilms formed by a mixture of strains exhibited a structure similar to individual bacterial biofilms but differed in polysaccharide composition. Additionally, lipids, comprising approximately 1.8% of the biofilm matrix were detected which were hypothesised to influence bacterial virulence and adherence. Moreover, the biofilm produced by *S. sanguis* demonstrated a higher protein content compared to the others, as indicated by an elevated absorbance intensity in the Amide I region, suggesting an increased potential for colonization. Limitations of this technique lies in its sensitivity to the scattering effect resulting in interfering signals, in addition to carbon dioxide (CO_2_) vibrational bands, necessitating replacing the CO_2_ peaks with linear trends, and data pre-processing, such as applying the Extended Multiplicative Scatter Correction (EMSC) method (Baker et al., 2018; Byrne et al., 2016). In addition, this method is very sensitive to water vapour, therefore, it requires thorough drying of the samples prior to FT-IR measurements. Otherwise, this could be overcome through water signal subtraction, or applying attenuated total reflectance (ATR) (Schmitt & Flemming, 1998). Furthermore, since it provides holistic information across the whole of the mid-IR spectrum, it requires applying validated and robust chemometrics for interpretation (Ellis & Goodacre, 2006).

### Metabolomics sample preparation pipeline

**Fig. 2:**
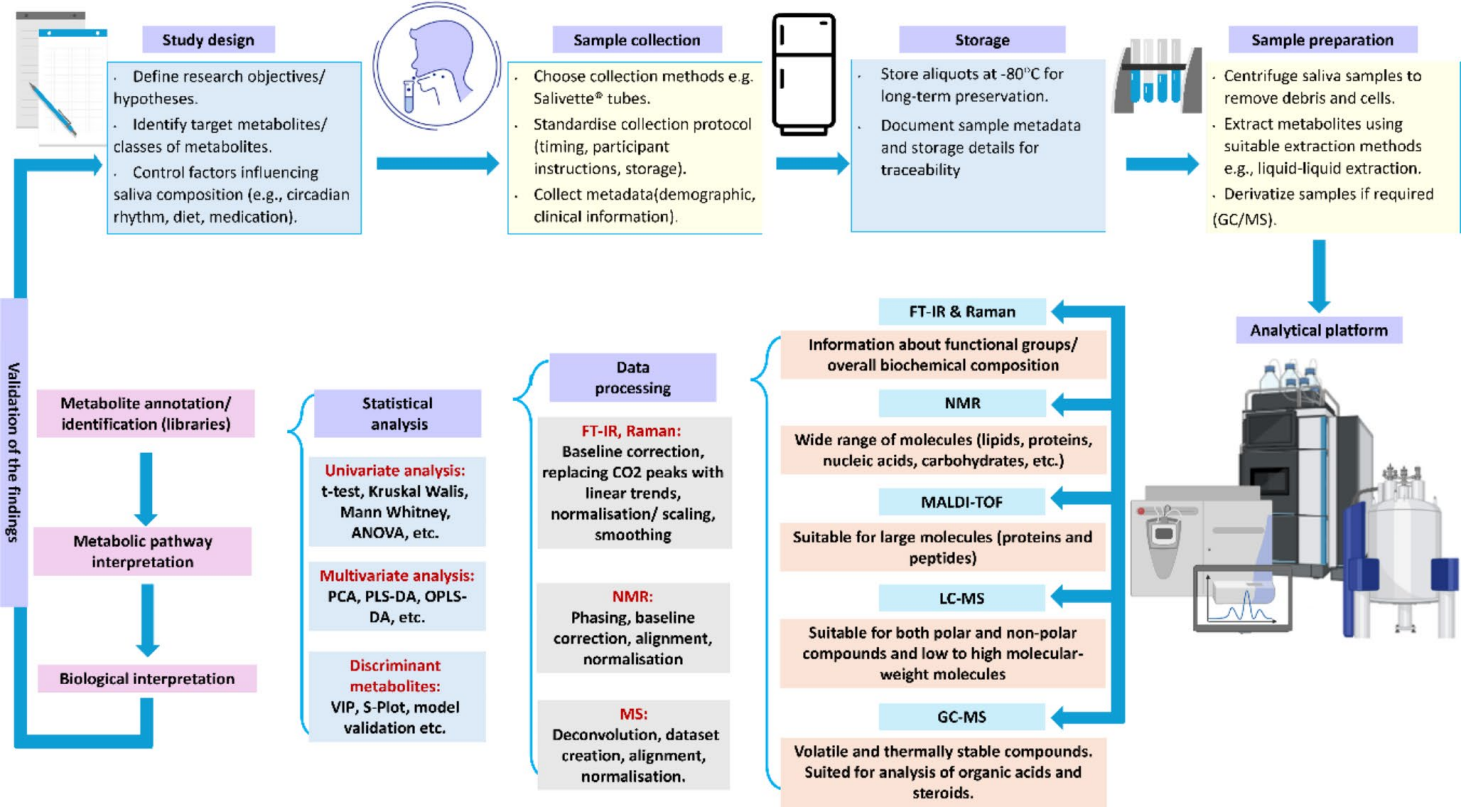
Salivary metabolomics experimental workflow from study design to analysis

The pre-analytical treatment of biological samples is a critical aspect of any metabolomic research, as it can influence the accuracy and reliability of the detected metabolite levels and consequently affect the overall quality of the data. This preparatory stage encompasses an array of operations, such as sample collection, quenching, extraction and storage. Sample preparation protocols play a pivotal role in preserving a snapshot of the metabolic profile of the biological system under investigation (Bruce et al., 2009). For human metabolomics studies focused on oral diseases, it is usually recommended that participants should be instructed not to eat, drink, chew gum, use oral hygiene, take medication or smoke before sample collection to avoid interference with the results (Bhattarai et al., 2018). Therefore, inappropriate sampling time and poor sample collection/preparation can dramatically decrease the accuracy and precision of the results (Tan et al., 2016). Another essential factor that should be considered is the metabolic flux, i.e., metabolites crossing through a reaction system over time, so that samples are not metabolically active after their collection point. This can occur since metabolites can undergo several biochemical reactions resulting in rapid metabolite turnover within seconds to minutes (Burgess et al., 2014). Sample quenching is crucial for stopping or slowing down such metabolic activities, to keep the metabolic profile constant during sample processing and it is usually carried out before extraction and at the point of collection (Sake et al., 2020). In order to minimise any potential changes post-sample collection, samples are usually flash-frozen in liquid nitrogen and stored at -80 °C immediately after sample collection (García-Villaescusa et al., 2018; Kuboniwa et al., 2016; Kumari et al., 2020). If -80 °C freezers are not available at the clinical site, freezing at -25 °C (Rzeznik et al., 2017), or collecting samples in sterile containers containing a preservative such as sodium azide and immediately freezing them in liquid nitrogen can be a sufficient alternative (Singh et al., 2017). Sample processing is typically determined by both the selected analytical platform for conducting the analysis and the type of metabolites of interest. Before carrying out the analysis, saliva samples should be thawed in ice, followed by centrifugation at low temperatures to remove any cell debris *(*Kumari et al., 2020; Romano et al., 2019; Rzeznik et al., 2017; Singh et al., 2017). Although saliva is an extracellular fluid, it is still considered metabolically active, due to the presence of various enzymes and oral microbiota, hence the samples should be kept on ice while being processed, similar to when processing plasma and serum (Chetwynd et al., 2017). For NMR analysis, saliva is typically subjected to buffering in a solution containing 0.2 mol/L phosphate buffer and subsequent dilution with deuterium oxide (Santone et al., 2014; Walsh et al., 2006). However, for MS analysis, metabolite extraction and separation techniques are essential (Gardner, Carpenter et al., 2020b). The untargeted approach, involves the extraction of most of the metabolites within the biological specimen in a manner that guarantees the reproducibility and accuracy of the results when the analysis is applied to a larger number of samples (Mathon et al., 2019), however, for a targeted approach the extraction process is tailored according to the metabolites of interest. In addition, these should include simple and quick steps to avoid changes of the metabolite-content of the samples during long preparation procedures (Vuckovic, 2012). Different extraction techniques have been reported for salivary metabolites depending on the type of the metabolites of interest, such as using cold methanol for thermolabile metabolites (Schulte et al., 2020), a mixture of methanol: water: chloroform (2.5:1:1) for extracting a wide range of polar to non-polar metabolites (Ozeki et al., 2016), or hexane for non-polar metabolites (Huang et al., 2014). An overview of the pipeline of the salivary metabolomics experiments is provided in (Fig. 2).

## Salivary metabolome

The salivary metabolome is known by its heterogeneity in terms of the metabolite chemistry and concentration. This complexity is exacerbated due to the contribution of various factors including the availability of nutrients (food intake), specific host demographics, health and lifestyle and the metabolism of the bacterial cells (Pozzi et al., 2022). In addition, the chemical composition of the saliva has been reported to be strongly affected by the collection method, gender, circadian clock, smoking status, nutrition, age, and health condition (Fischer & Ship, 1999; Larsen et al., 1999). Some metabolites, such as acetate and propionate, were found to be elevated if the patient’s sample was collected before breakfast, whereas their concentrations after lunch were much lower (Dame et al., 2015). Apart from sleeping or eating, amino acid concentrations such as L-leucine, L-alanine, betaine and glycine, have also been shown to vary depending on the rhythms of the circadian clock (Dallmann et al., 2012; Dame et al., 2015).

Studies have reported that salivary metabolites span approximately about 121 chemical classes, such as carboxylic acids, steroids, quaternary ammonium salts and others (Dame et al., 2015; Wishart et al., 2008). Hence, it is crucial to determine the source of the metabolite detected in saliva or GCF and link them to their origin, such as the host, external factors, or oral microbiota. A few studies have tried to discriminate oral metabolites for example short-chain fatty acids (SCFAs) such as acetate, butyrate and propionate and correlate them to oral bacterial production (Gardner et al., 2019). However, studying the link between the oral microbiome and the salivary metabolome is still in its infancy (Gardner, So et al., 2020a). Since the metabolome is constantly changing and any given profile is a snapshot of the gene and protein expressions, metabolomics has the potential to produce quantitative data and be used as a tool to clarify the link between the metabolome dynamic and illness status (Gorr, 2012).

### Key metabolites in saliva

Several salivary metabolomics studies have used approaches to define the significance of key metabolites which may be utilised as biomarkers of systemic diseases. Interestingly, a number of systemic diseases have been shown to have an association with chronic periodontitis. There are a range of different analytical methods to detect potential biomarkers found in chronic periodontitis patients which have been isolated from GCF or saliva samples (Table 3).

**Table 3:** Salivary metabolomics studies to investigate chronic periodontitis and systemic disease biomarkers

Metabolomics studies of indicative biomarkers of systemic diseases that might be associated with chronic periodontitis					
**Systemic disease**	**Type of saliva**	**Method**	**Biomarker metabolites**		**Reference**
			**↑↑↑**	**↓↓↓**	
Oral cancer	OW	LC-MS/MS, LC-MS, GC-MS	2-Hydroxyglutarate 3-Glycosyl tryptophan Adenosine-3-monophosphate Guanosine-3-monophosphate N-acetylputrescine Spermidine Uracil	NM	(Mukherjee et al., 2017)
	WS	CE-MS	2-hydroxy-4-methylvaleric acid 2-oxoisovaleric acid 3-(4-hydroxyphenyl) propionic acid 3-hydroxybutyric acid 3-phenyllactic acid 3-phenylpropionic acid Alanine Butyric acid Cadaverine Choline Glycolic acid Heptanoic acid Hexanoic acid hydroxyphenylacetic acid Isoleucine Leucine Octanoic acid Taurine Terephthalic acid Trimethyllysine Tryptophan Valine γ-butyrobetaine	Urea	(Ohshima et al., 2017)
	WS	^1^H-NMR, LC-MS/MS	NM	Glycine Proline	(Lohavanichbutr et al., 2018)
Alzheimer’s disease	OW	^1^H-NMR	Acetone Propionate	NM	(Yilmaz et al., 2017)
	WS	LC-MS	Choline-cytidine Histidinyl-Phenylalanine, Methylguanosine	NM	(Huan et al., 2018)
	WS	(DFI-MS/MS)	NM	Acyl-alkyl phosphatidylcholines	(Marksteiner et al., 2019)
Parkinson’s disease	USWS	^1^H-NMR	Acetate Acetoin Alanine Fucose GABA Glycine Histidine Isoleucine Lysine N-acetyl groups Phenylalanine Propionate Trimethylamine, Trimethylamine-oxide Tyrosine Valine	NM	(Kumari et al., 2020)
Diabetes	NM	GC/MS, LC/MS	Adenosine Arachidonate(20:4n6) Palmitoyl sphingomyelin Cysteine-glutathione disulfide 12-HETE Docosapentaenoate(n3 DPA; 22:5n3) Glutathione oxidised (GSSG) Guanine Guanosine Inosine Linoleate (18:2n6) Linolenate [α or γ; (18:3n3 or 6)] Xanthine	NM	(Barnes et al., 2014)
	USWS	^1^H-NMR	Acetate Lactate Sucrose	Succinic acid	(de Oliveira et al., 2016)
**Oral biofluids metabolomic analysis for indicative biomarkers of chronic periodontitis**					
**Oral biofluid**		**Method**	**Indicative biomarkers**		**Reference**
			**↑↑↑**	**↓↓↓**	
OW and tongue swab		^1^H-NMR	Acetone Isopropanol Lactic acid Taurine	Glycerol Methanol	(Gawron et al., 2019)
WS		GC-TOF/MS	5-oxoproline Cadaverine Histidine	NM	(Kuboniwa et al., 2016)
GCF		GC-MS	3TMS derivative 5-aminovaleric acid Serine	NM	(Rodrigues et al., 2021
USWS		^1^H-NMR	Isoleucine Phenylalanine Proline Tyrosine Valine	Lactate Pyruvate	(Romano et al., 2019)
WS		^1^H-NMR	4-aminobutyrate Butyrate Caproate Choline Isocaproate Isoleucine Isopropanol Isovalerate Methanol	Lactate Proline Sucrose Sucrose-glucose-lysine	(García-Villaescusa et al., 2018)
USWS		^1^H-NMR	Alanine Glutamate Lactate Leucine Pyruvate Succinate Valine	NM	(Singh et al., 2017)

↑↑↑= upregulated metabolites, ↓↓↓= downregulated metabolites, OW = oral washout, WS = Whole saliva, USWS = unstimulated whole saliva, NM = not mentioned, LC-MS/MS = liquid chromatography with tandem mass spectrometry, LC-MS = liquid chromatography mass spectrometry, GC-MS = gas chromatography mass spectrometry, ^1^H-NMR = proton nuclear magnetic resonance spectroscopy, DFI-MS/MS = Flow injection analysis- tandem mass spectrometry

GC-TOF/MS = gas chromatography- time of flight mass spectrometry. GCF = gingival crevicular fluid

### Salivary metabolites indicative of systemic disease

While saliva offers notable advantages in terms of sample collection and preparation compared to other human biofluids such as cerebrospinal fluid and blood, the field of metabolic profiling in saliva is in its infancy compared to studies conducted on urine and plasma (Gardner et al., 2018). However, in the context of periodontitis, the majority of salivary metabolomics studies have investigated saliva as a diagnostic tool to discern differences in metabolite levels among individuals already diagnosed with periodontitis. Among these studies, Kim et al. applied untargeted metabolomic analysis using ^1^H-NMR to analyse the salvia of 221 participants to compare the metabolic profiles of patients with periodontitis to a control group, and found that five metabolites, ethanol, taurine, isovalerate, butyrate, and glucose could be used as potentially promising biomarker candidates for periodontitis diagnosis (Kim et al., 2021).

### Chronic periodontitis and kidney disease

The comorbidity of CKD and chronic periodontitis has been investigated in multiple studies (Lapo et al., 2021; Serni et al., 2023). It has been suggested to be a bidirectional relationship and this could be manifested in two ways. Firstly, the periodontium in chronic periodontitis acts as a pocket for several bacteria that can form biofilms. Due to the rich blood vessels that line the oral cavity, this would also facilitate the continuous spreading of the periodontal pathogens and their metabolites into the bloodstream, causing bacteriemia. Through this route, pathogens and their toxins can be carried into distal places of the body, such as the kidneys, potentially leading to impaired kidney function (Kshirsagar et al., 2007). It has further been suggested that the presence of chronic periodontitis could agitate a systemic inflammatory response, which would promote the permeability of the blood vessels in kidneys, potentially leading to proteinuria and renal thrombosis leading to diminished renal function (Kitamura et al., 2019). In contrast, some patients with acute kidney disease or at the end stage of chronic kidney disease might suffer from uraemia, which could dysregulate the immune system and enhance infection vulnerability, such as in the case of chronic periodontitis (Lapo et al., 2021). *P. gingivalis* has been implicated in the development of chronic kidney disease through its capacity to disrupt the immune system. This has been shown to occur via the activation of complement 3 (C3) receptors on monocytes by the fimbriae proteins of *P. gingivalis*, leading to sustained inflammation in glomerular endothelial cells, which can promote glomerular sclerosis (Chou et al., 2005; Fisher et al., 2008; França et al., 2017). Additionally, gingipains from *P. gingivalis* were found to activate the toll-like receptor 2-phosphoinositide-3 kinase signalling pathway, contributing to renal fibrosis (França et al., 2017). In terms of chronic periodontitis, it can be clinically diagnosed by a number of examinations, such as probing pocket depth, bleeding on probing, and radiography (Kuboniwa et al., 2016). However, such clinical measurements have limitations as they are time-consuming, non-quantitative and cannot distinguish the disease activity as they sometimes indicate previous periodontitis activity (Ji & Choi, 2015). Moreover, since in the initial stages, painful symptoms are not usually manifested, it is rarely diagnosed in its early stages. Most patients are diagnosed with chronic periodontitis after progression into its severe forms making the timely diagnosis and appropriate treatment of chronic periodontitis challenging (Shaddox & Walker, 2010). This emphasises the necessity for the development of quick, safe and easy methods to diagnose chronic periodontitis in its early stages.

The high morbidity and mortality rates of kidney disease and its impact on the quality of life of patients further necessitate the investigation of diagnostic methods that enable the detection of kidney disease in its early stages, with the aim of treating the condition before its deterioration. There has been increasing evidence of links between the oral microbiome and kidney disease. Furthermore, for studying the aetiology of chronic periodontitis, previous studies investigated the salivary and GCF to highlight indicative biomarkers for chronic periodontitis (Gawron et al., 2019). Therefore, the opportunity to use saliva as a diagnostic tool for chronic periodontitis has been emerging over the last decade as it is considered the optimal biofluid in terms of its simple, safe, quick, and painless collection method (Taylor, 2014). Following this concept, other researchers have also reported the use of salivary metabolomics to monitor oral health status and diagnose periodontal diseases such as dental caries (Fidalgo et al., 2013) and oral cancer (Al-Hebshi et al., 2014).

These findings highlight the potential of metabolomics approaches for the detection and identification of chronic periodontitis-associated bacterial metabolites within such oral biofluids with the purpose of early diagnosis of idiopathic systemic illnesses.

### Periodontal markers and diabetes

Since most of the metabolites present in blood can be found in saliva (Spielmann & Wong, 2011; Takeda et al., 2009), several previous studies have also explored the idea of using salivary metabolites as indicative biomarkers for systemic diseases. Using salivary metabolomics as a diagnostic tool has expanded from the detection of oral diseases to identifying other physiological conditions. In diabetes patients, Barnes et al. conducted a cross-sectional study involving 161 participants categorized into either type 2 diabetes or healthy groups (Barnes et al., 2014). Subsequently, the participants were further stratified based on their periodontal health status, resulting in subgroups classified as those with a healthy oral environment, gingivitis or periodontal disease. Comprehensive untargeted metabolomics analyses were performed on whole saliva samples using both GC-MS and LC-MS techniques, in both positive and negative modes, followed by ANOVA and t-tests, which were employed for data comparison. The study indicated a substantial elevation in the levels of purine degradation products within saliva including adenosine, cysteine-glutathione disulfide, guanine, guanosine, inosine and xanthine. Additionally, elevated levels of fatty acids such as 12-HETE, linoleate (18:2n6), linolenate [α or γ; (18:3n3 or 6)], docosapentaenoate (n3 DPA; 22:5n3), arachidonate (20:4n6) and palmitoyl sphingomyelin were observed in the saliva samples of those patients with Type 2 diabetes.

A separate investigation targeting children was undertaken by de Oliveira et al. employing 1 H-NMR, this study analysed the entire saliva composition of 68 children aged 2 to 6 years, categorizing them into two groups: those with type 1 diabetes and a control group (de Oliveira et al., 2016). Subsequently, PLS-DA was applied to compare the salivary profiles between the two groups. The findings of the study revealed the upregulation of four potential biomarkers including acetate, n-acetyl-sugar, lactate and sugar in the diabetes 1 group, along with the downregulation of succinic acid when compared to the salivary composition of healthy children.

### Periodontal disease and Alzheimer’s disease

In a study conducted by Dominy et al., an association between *P. gingivalis* and Alzheimer’s disease was established (Dominy et al., 2019). The findings of this study revealed that oral infection with *P. gingivalis* correlated with Alzheimer’s disease progression, as evidenced by the proportional relationship between its levels in saliva and cerebrospinal fluid (CSF). The release of cystine protease gingipains such as lysine-gingipain (Kgp), arginine-gingipain A (RgpA), and arginine-gingipain B (RgpB), was found to escalate neural damage. However, the application of a specifically designed gingipain-inhibitor demonstrated a reduction in neuroinflammation and a decrease in the production of amyloid beta Aβ_1–42_, a peptide component of amyloid plaques in the Alzheimer’s brain. In the diagnostic assessment of Alzheimer’s disease, Huan et al. employed HPLC-MS with an electrospray ionization (ESI) interface operating in positive ion mode (Huan et al., 2018). This approach was utilised to profile the amine/phenol sub-metabolome in saliva samples obtained from 109 participants categorized into three groups: Alzheimer’s disease, mild cognitive impairment, and cognitively normal individuals. To enhance the detection and ionization of phenols and amines, the samples were isotopically labelled with dansyl chloride. The results revealed higher levels of three metabolites, namely methylguanosine, histidinyl-phenylalanine, and choline-cytidine, in the saliva of individuals with Alzheimer’s disease compared to those in the cognitively normal group. Additionally, elevated levels of amino-dihydroxybenzene, glucosylgalactosyl hydroxylysine - H_2_O, and aminobutyric acid + H_2_ were observed in the Alzheimer’s disease group compared to individuals with mild cognitive impairment.

### Summary of bacteria and salivary metabolites linked chronic periodontitis with systemic diseases

As evidenced, many metabolites produced from bacteria associated with periodontal disease have been linked to systemic diseases. Interestingly, there are differences in the oral bacteria and metabolic profiles found in saliva associated with the different diseases. Among chronic periodontitis-associated bacteria, *P. gingivalis* is the most frequently reported oral microbial candidate in oral cancer (Lim et al., 2018), Alzheimer’s disease (Maurer et al., 2018), Parkinson’s disease (Olsen et al., 2020), chronic kidney disease (Bastos et al., 2011), and diabetes (Aemaimanan et al., 2013). Furthermore, apart from oral cancer and Parkinson’s disease, all the above-mentioned diseases were found to be linked with *T. forsythia* and *T. denticola* (Bastos et al., 2011; Leblhuber et al., 2020). However, *F. nucleatum* was reported only with Alzheimer’s disease (Maurer et al., 2018). In terms of the salivary biomarker metabolites, it has been found that alanine, cadaverine, choline, isoleucine, leucine and taurine were upregulated in oral cancer and chronic periodontitis while proline was found downregulated in both of them (Gawron et al., 2019; Kuboniwa et al., 2016; Ohshima et al., 2017; Romano et al., 2019; Singh et al., 2017). Moreover, histidine, phenylalanine, and tyrosine were reported as upregulated salivary metabolites in both Parkinson’s disease and chronic periodontitis (Kuboniwa et al., 2016; Kumari et al., 2020; Romano et al., 2019). In addition, alanine, isoleucine, and valine are three metabolites that have also been highlighted as being upregulated in oral cancer, Parkinson’s disease, and chronic periodontitis (García-Villaescusa et al., 2018; Kumari et al., 2020; Ohshima et al., 2017; Yilmaz et al., 2017). On the other hand, acetone was found to be upregulated in both Alzheimer’s disease (Yilmaz et al., 2017) and chronic periodontitis (Gawron et al., 2019), whereas lactate was reported to be an upregulated salivary metabolite in both types of diabetes (de Oliveira et al., 2016) and chronic periodontitis (Singh et al., 2017). Therefore, it can be concluded that an array of metabolites may be indicative of specific systemic diseases that occur in periodontal patients.

## Future directions

There is a remarkable bidirectional relationship between chronic periodontitis and other systemic illnesses that have been reported in several previous studies. It is noteworthy that the metabolites identified within GCF exhibit dissimilar profiles when compared to those within saliva. Furthermore, the composition of the salivary metabolome is influenced by a multitude of variables, and failure to control for these factors can introduce substantial variability. To date, the primary focus of research has been directed toward investigating chronic periodontitis-associated pathogens as potential biomarkers for specific systemic diseases. A limited number of studies have also explored the use of polyamines, including putrescine, cadaverine, spermidine and spermine, originating from bacteria, as potential biomarkers for a variety of diseases. However, it is crucial to undertake more comprehensive research to thoroughly investigate the salivary metabolites produced by periodontal bacterial communities within the context of diagnosing systemic diseases. Assessment of such bacteria in simulated environments, such as artificial saliva or GCF, that mimics the oral environment and growth conditions, serves as a foundational approach to gain deeper insights into their metabolic products. These findings can then be validated through more targeted analysis of samples collected from actual patient cohorts. Furthermore, the integration of salivary multi-omics approaches, including genomics, transcriptomics, proteomics, and metabolomics holds the potential to significantly advance our understanding of the biological dynamics underpinning chronic periodontitis biofilms, while offering a deeper understanding of the oral biofluid constituents, and guiding the detection and identification of potential biomarkers. These findings may also serve as a fundamental basis and streamline the process of identifying specific target metabolites for the purpose of developing biosensors, which can be subsequently utilised for the diagnosis and monitoring of various diseases, offering an enhanced level of convenience, sensitivity, and reliability in these clinical applications.

## Data Availability

No datasets were generated or analysed during the current study.
